# Contribution of Genetic Polymorphisms of Inflammation Response Genes on Sporadic Colorectal Cancer Predisposition Risk in Malaysian Patients – A Case Control Study

**DOI:** 10.31557/APJCP.2019.20.6.1621

**Published:** 2019

**Authors:** Ravindran Ankathil, Mohd Aminudin Mustapha, Ahmad Aizat Abdul Aziz, Siti Nurfatimah Mohd Shahpudin, Andee Dzulkarnaen Zakaria, Muhammad Radzi Abu Hassan, Kamarul Imran Musa

**Affiliations:** 1 *Human Genome Centre, *; 3 *Department of Surgery, *; 5 *Department of Community Medicine, School of Medical Sciences, Universiti Sains Malaysia, Health Campus, Kubang Kerian, Kelantan,*; 2 *Center of Pre University Study, Universiti Malaysia Sarawak, Kota Samarahan, Sarawak, *; 4 *Hospital Sultana Bahiya, Alor Setar, Kedah, Malaysia. *

**Keywords:** Colorectal cancer, inflammatory response genes, polymorphisms

## Abstract

**AIM:**

To investigate the frequencies and association of polymorphic genotypes of *IL-8* -251 T>A, *TNF-α *-308 G>A, *ICAM-1* K469E, *ICAM-1* R241G, *IL-6* -174 G>C, and *PPAR-γ* 34 C>G in modulating susceptibility risk in Malaysian colorectal cancer (CRC) patients.

**Methods:**

In this case-control study, peripheral blood samples of 560 study subjects (280 CRC patients and 280 controls) were collected, DNA extracted and genotyped using PCR-RFLP and Allele Specific PCR. The association between polymorphic genotype and CRC susceptibility risk was determined using Logistic Regression analysis deriving Odds ratio (OR) and 95% CI.

**Results:**

On comparing the frequencies of genotypes of all single nucleotide polymorphisms ( SNPs ) in patients and controls, the homozygous variant genotypes *IL-8* -251 AA and *TNF-α* -308 AA and variant A alleles were significantly higher in CRC patients. Investigation on the association of the variant alleles and genotypes singly, with susceptibility risk showed the homozygous variant A alleles and genotypes *IL-8* -251 AA and *TNF-α* -308 AA to be at higher risk for CRC predisposition. Analysis based on age, gender and smoking habits showed that the polymorphisms *IL8 *-251 T>A and *TNF – α* 308 G>A contribute to a significantly higher risk among male and female who are more than 50 years and for smokers in this population.

**Conclusion:**

We observed an association between variant allele and genotypes of *IL-8*-251 T>A and *TNF-α*-308 G>A polymorphisms and CRC susceptibility risk in Malaysian patients. These two SNPs in inflammatory response genes which undoubtedly contribute to individual risks to CRC susceptibility may be considered as potential genetic predisposition factors for CRC in Malaysian population.

## Introduction

With more than one million new cases and half a million deaths every year worldwide, Colorectal cancer (CRC) is a major public health problem, in developed as well as in developing countries (Ferlay et al., 2010). CRC is a multifactorial disease and its onset is attributed to complex interactions between environmental, and host’s genetic predisposition factors. In addition to several environmental factors including life style habits and dietary factors, obesity, inflammation and diabetes are also risk factors for CRC (Yehuda-Shnaidman and Schwartz, 2012). Biological, animal, clinical and epidemiological studies had indicated a clear association between CRC and inflammation (Coussens and Werb, 2002; Wang and DuBois, 2013; Nieminen et al., 2013). Patients with inflammatory bowel disease (IBD) including Crohn’s disease (CD) and ulcerative colitis (UC) have an increased risk of CRC (Nieminen et al., 2013). Approximately 1:6 individuals with IBD have been reported to develop CRC (Lakatos and Lakatos, 2007). Genetic factors, especially those associated with inflammation have been strongly suggested to be involved in IBD development and an association between inflammatory response genes and IBD development has been established (Garrity-Park et al., 2012). Chronic inflammation is a common underlying cause in the development of many gastrointestinal cancers including CRC and is considered as a predisposing factor for malignant transformation. Despite several evidences strongly implicating chronic inflammation as a culprit in colorectal carcinogenesis, surprisingly, little research has directly addressed the genetic predisposing factors which mediate inflammatory response and favors CRC development. Since inflammation constitutes one of the molecular networks underlying susceptibility to IBD and CRC, it was hypothesized that genes which mediate inflammatory response might be a group of candidate genes for CRC predisposition.

The genetic constitution of an individual also has been hypothesized to play an important role in sporadic CRC predisposition. A great deal of attention has been focused in the field of genetic variations in the host as factors contributing to cancer predisposition. Genetic polymorphisms (single nucleotide polymorphisms) have emerged in recent years as important determinants of disease susceptibility and severity. It is well documented that genetic variations, especially single nucleotide polymorphisms (SNPs) in genes involved in several biological pathways, play a key role in determining how individuals respond at the cellular level to various environmental conditions including inflammation. Polymorphisms (SNPs) of several cytokine genes have emerged as enhancers of susceptibility and severity of gastrointestinal malignancies (Macarthur et al., 2004). Researchers had identified several polymorphisms in regulatory regions of cytokine genes that could contribute to cancer progression (Hwang et al., 2002, Gunter., et al 2006, Theodoropaulos et al., 2006, Wilkening et al., 2008). SNPs that occur in cytokines gene even in the promoter regions or other regulatory regions have also been reported to influence the development and the progression of CRC (Wilkening et al., 2008). Few genes such as interleukin 6 (*IL-6*), interleukin 8 (*IL-8*), tumour necrosis factor alpha (TNF-α), intercellular adhesion molecule 1(*ICAM-1*) and also peroxisome proliferator-activated receptor gamma (*PPAR-γ*) were selected as candidate genes for the present study as they are known to be important for inflammation of colorectum and their allelic variants have been shown to have biological effects. 

*TNF-α*, located on chromosome 6 (6 p21.33), is a pro-inflammatory, pleiotropic cytokine which has been implicated in the pathogenesis and progression of various cancers. The *TNF-α*-308 G>A polymorphism (rs 1800629) which involves a substitution of guanine (G) to adenine (A) at -308 bp position, is located within the 5′ flanking region which is a regulatory hot spot region of TNF. ICAM1 is a transmembrane glycoprotein belonging to the immunoglobulin superfamily of cell adhesion molecules, normally expressed on the surface of various types of cells: leucocytes, endothelial cells and fibroblasts (Yang et al., 2005; Hayes and Seigel, 2009). The *ICAM1*, located on chromosome 19p13, has at least two functional bi-allelic polymorphisms, one at codon 241(glycine to arginine substitution, G>A; rs179969) in exon 4 and another one at codon 469 (a lysine to glutamic acid substitution; A>G; rs5498) in exon 6. On chromosome 3p25 is located *PPAR-γ* which is a member of the nuclear hormone receptor super family that plays a pivotal role in regulating adipocyte differentiation, lipid and glucose metabolism, insulin insensitivity and immune response (Thompson, 2007). A common structural polymorphism is 34 C>G (rs1801282) located at codon 12 (Pro12Ala) of *PPAR-γ2* specific exon 8. *IL-6* is a pleiotropic cytokine located on chromosome 7 p21-24 that plays a central role in haematopoiesis, as well as in the differentiation and growth of a number of cells of different histologic origin such as endothelial cells, keratinocytes, neuronal cells, osteoblasts and osteoclasts (Yamin et al., 2008). In human *IL-6* gene, the polymorphism that change guanine to cytosine at the -174 promoter region (*IL-6*-174 G>C; rs1800795) has been reported to increase IL-6 levels (Bonafe et al., 2001). *IL-8* (located on chromosome 4q13-q21) is a pro-angiogenic chemokine and one of the major mediators of inflammatory response. Of the several polymorphisms detected in *IL-8*, a common polymorphism at -251 position (*IL8*- 251 T>A; rs4073) of the promoter region has been associated with transcription activity of the gene (Hull et al., 2000). We aimed to investigate the contribution of the polymorphisms of the above mentioned inflammatory response genes as predisposition risk factors for CRC susceptibility in Malaysian population. 

As a first step, we conducted a case-control study (involving 255 CRC cases and 255 normal controls) to investigate the association of *IL-8*-251 T>A polymorphism singly on sporadic CRC susceptibility risk. Variant allele and genotype of *IL-8*-251 T>A was found to be significantly associated with CRC susceptibility risk (Mustapha et al., 2012). In the light of these results, we extended the case-control study on more number of subjects and investigated the association of few additional polymorphisms of inflammatory response genes with CRC predisposition risk. This report encompasses the genetic association study carried out on the association of inflammatory response genes polymorphisms such as *TNF-α* -308 G>A, *ICAM-1* K469E, *ICAM-1* R241G , *IL-6 *-174 G>C , *PPAR-γ* 34 C>G, and also including *IL-8* -251 T>A, with susceptibility risk in Malaysian CRC patients. The previously reported SNP *IL-8*-251 T>A (Mustapha et al., 2012) (17) was also considered in the present analysis after adding on more study subjects. 

## Materials and Methods

*Study setting and study subjects*

This study was carried out in Human Genome Centre, Universiti Sains Malaysia, Malaysia, after getting approval from Research Review Board and Research Ethics Committee (Human) of Universiti Sains Malaysia (Ref. no. USMKK/PPP/JEPeM [(208.4(1.4)]) and Ethical approval from Ministry of Health, Malaysia (Registration ID. NMRR-09-351-4033). For this case control study, CRC patients were recruited from Hospital Universiti Sains Malaysia (HUSM) and few hospitals under the Ministry of Health, Malaysia such as Hospital Sultanah Bahiyah, Alor Setar, Kedah and Hospital Raja Perempuan Zainab II, Kota Bharu, Malaysia.

*Inclusion criteria*

Sporadic colorectal cancer cases were identified from the database of pathology laboratories that reviewed all histologies. For inclusion, cases were histo-pathologically confirmed colorectal cases, who did not have previous colon/rectal or other cancers, and were between 30 and 70 years age at diagnosis and who were sufficiently mentally competent to participate in the interview on collection of personal and other epidemiological data. All procedures followed the ethical standards of institutional and national committee on human experimentation and the 2008 revision of 1975 Declaration of Helsinki.

*Exclusion criteria*

Cases with known (as indicated in the pathology reports) familial adenomatous polyposis (FAP), Hereditary Nonpolyposis Colorectal Cancer (HNPCC) or any other previous malignancy were excluded. Those patients with a history of systemic diseases of immune-inflammation mechanism were excluded from the study. 

*Controls*

Controls were normal healthy individuals, volunteers who visited HUSM or other hospitals, for other problems unrelated to colorectal or other cancers. Controls were genetically unrelated to the cases and were without individual history of cancer and inflammatory diseases. 

*DNA extraction*

Peripheral blood samples of 280 normal controls and 280 clinically diagnosed and histo-pathologically confirmed CRC patients were collected in EDTA tubes, after obtaining written informed consent. The collected samples were stored at -20^o^C till use. Genomic DNA was extracted using commercial DNA extraction kit (QIAGEN, Hilden, Germany) and the genes of interest were amplified using appropriate primers.

*Genotyping*

Out of the six SNPs included in this study, four SNPs (*TNF-α* -308 G>A, *ICAM-1* K469E, *IL-6* -174 G>C and *PPAR-γ* 34 C>G) were genotyped using Polymerase Chain Reaction-Restriction Fragment Length Polymorphism (PCR-RFLP) technique. The other 2 SNPs (*IL-8* -251 T>A and *ICAM-1* R241G) were genotyped using Allele specific PCR.

*DNA sequencing*

As part of quality control, and for confirmation of the genotypes, 10 % of random samples representing all the SNPs were chosen and were sent for DNA sequencing (First Base Laboratory Sdn Bhd, Malaysia), and 100% concordance rate was achieved. 

*Statistical analysis*

Statistical analysis was carried out using SPSS version 20. Both cases and controls were analyzed with a Chi square test to determine whether they were in Hardy-Weinberg equilibrium. The differences in distribution of various genotype and allele frequencies of the SNPs studied, gender and age between the cases and controls were calculated using Chi square, χ^2^ test. The associations of polymorphic genotypes with CRC susceptibility risk was determined by using logistic regression analysis deriving odds ratios (ORs) and the corresponding 95% confidence intervals (CI). The ORs of CRC risk associated with the variant allele carrier and homozygous variant genotype for each of the polymorphism was based on the homozygosity of the major allele as the reference group. The base line group was the common major allele homozygote which by definition has an OR of 1. An association with a p-value of less than 0.05 was considered as significant. 

## Results

*Distribution of study subjects based on gender and age*

Altogether 560 study subjects (280 cases and 280 normal controls) were recruited in this case-control study. Among the study subjects, cases consisted of histo-pathologically confirmed CRC patients and controls comprised of healthy normal volunteers. Table 1 shows the distribution of study subjects based on gender and age. Out of the 280 CRC patients, 140 (50 %) were females and 140 (50%) were males. In the case of controls also, out of 280, 140 (50%) were females and 140 (50 %) were males. The mean age was 53.17 + 7.07 years for the cases and 46.47+12.02 years for the controls. There was significant difference in the incidence of CRC between two age groups (p-value <0.001). Sporadic CRC incidence was higher among individuals more than 50 years compared to those less than 50 years. 

*Genotypes frequencies of inflammation response genes in study subjects*

The results of the Hardy Weinberg equilibrium analysis of each SNP for both cases and controls showed values >0.05 (data not shown). This indicated that the cohort was in HWE and suggested that the results were reliable. Table 2 summarizes the results on the frequencies of the genotypes and alleles of the six SNPS studied in cases and controls. For *TNF-α* -308G>A, the frequencies of homozygous wild type -308 TT genotype was significantly higher in normal controls (p=0.005), whereas the homozygous variant genotype *TNF-α* -308 AA was significantly higher (p<0.001) in CRC patients compared to controls. The variant allele frequency was significantly higher among CRC patients (p=0.001). Comparison of the genotype frequencies *ICAM-1*K469E polymorphism between patients and controls showed no significant difference in the genotype and allele frequencies between them. For *ICAM-1* R241G polymorphism, the frequencies of homozygous wild type R241R was significantly higher in controls compared to CRC patients, with p value of 0.045. With regard to allele frequencies among controls, only major allele (R) was observed (100%) and no variant allele was observed. There was no significant difference in the frequencies of alleles and genotypes between cases and controls for *IL-6* - 174 G>C single nucleotide polymorphism. In the case of *PPAR-γ* 34 C>G, the frequency of homozygous wild type CC and wild type allele (C) were higher in controls (p=0.030 and p=0.031 respectively), as shown in Table 2. On comparing the frequencies of *IL-8* -251T>A genotypes of patients and controls, the homozygous variant -251AA was significantly higher in CRC patients (p=0.001) and the frequency of variant allele A of *IL-8* was also significantly higher in CRC patients (p=0.041). 

*Risk association of inflammation response gene polymorphisms with CRC susceptibility*

After determining the frequencies of the different categories of genotypes for each of the 6 SNPs included in the study, the association of the variant genotypes with CRC susceptibility risk was evaluated and the results are shown in Table 3. *TNF-α* homozygous variant genotype (*TNF-α* -308 AA) showed significantly higher risk for CRC development with OR 5.484 (CI 2.327 – 12.924, p<0.001). Allele A of *TNF-α* -308 was associated with increased risk of CRC, since it was found over-represented among CRC patients (34.4% vs 24.3%, p<0.001, OR 1.627, 95% CI: 1.254 – 2.110). The variant genotypes (heterozygous and homozygous variant) of *ICAM-1* K469E did not show significant association for CRC susceptibility risk. Also, the variant allele (G) of *ICAM-1* did not show any higher risk for CRC susceptibility. The risk association of *ICAM-1* R241G with CRC susceptibility risk could not be evaluated, due to 0% of heterozygous and homozygous variant allele and genotype frequencies, in controls. The heterozygous variant genotype of *IL-6* -174 G>C did not show any significant association with CRC susceptibility risk and the risk of homozygous variant genotype could not be evaluated, due to 0% frequency of this genotype in control samples. The variant allele also did not show significant risk for CRC susceptibility. The homozygous variant genotype of *PPAR-γ* 34GG was not present in any of the CRC patients and controls. In all the normal control samples, only homozygous wild type genotype was observed. So, the risk association of *PPAR-γ* 34 C>G with CRC susceptibility risk could not be evaluated, due to 0% of heterozygous and homozygous variant genotype frequencies. The risk association of the allele of *PPAR-γ* polymorphism could not be assessed due to the presence of only wild type alleles in control sample. In the case of *IL-8*, the homozygous variant genotype (*IL-8* -251AA) showed significantly higher risk for CRC susceptibility with OR 4.133 (CI 1.937 – 8.18, P=0.001). The variant allele (A) of *IL-8* also showed significantly higher risk with OR 1.345, (CI 1.062-1.704, p=0.013).

*Risk association based on gender stratification*

We stratified our study subjects according to gender and investigated the association of the all candidate polymorphisms with colorectal cancer susceptibility risk and the results are shown in Table 4. The *TNF-α *homozygous variant genotype (*TNF-α* -308 AA) conferred a higher risk for CRC development in female subjects (OR 7.661, 95% CI 2.166-27.098, p= 0.002). Likewise, the homozygous variant genotype of *IL-8* -251AA also showed significantly higher risk among females with OR 11.429 (CI 2.372-55.054, P=0.002). The other variant polymorphisms did not show any significant gender specific association with CRC risk.

*Risk association based on age group*

Risk associations based on age groups are shown in Table 5. The homozygous variant genotype AA of *IL-8* was associated with higher risk for CRC development in both age groups – for individuals in the more than 50 years age group (OR 3.733, 95% CI 1.276-10.919, p=0.016) as well as those in less than 50 years age group (OR 8.095, 95% CI 2.209-29.660, p=0.002). However, the variant genotype AA of *TNF-α* showed higher CRC risk association only in those above 50 years age group (OR 19.440, 95% CI 2.582-146.367, p=0.004 ). No significant association between age group and CRC risk was observed for other polymorphisms studied. 

*Risk association based on smoking status stratification*

When the results were stratified according to smoking status, (as shown in Table 6), the homozygous variant genotype of *IL-8* -251AA showed significantly higher risk with OR 3.664 (CI 1.407-9.538, P=0.008) in smokers. In the case of *TNF-α* -308 G>A polymorphism, the heterozygous genotype (OR 1.690, 95 % CI 1.101-2.593, p=0.016) and homozygous variant genotype (OR 7.358, 95% CI 2.847-19.014, p=0.001) showed higher risk in smokers. The other polymorphisms, failed to show any association with CRC risk when stratified based on smoking status of the study subjects. 

## Discussion

In this Malaysian case-control study, we investigated the association of few candidate SNPs in the inflammation related pathway, with sporadic CRC predisposition risk. We observed association of polymorphisms of inflammatory response genes *TNF-α* -308 G>A and *IL-8* -251T>A with higher risk for CRC susceptibility among Malaysian patients. Polymorphisms of inflammation related genes have been investigated for their association with CRC in few other populations, but results are inconsistent. However, reports are unavailable for Malaysian CRC patients. To the best of available knowledge, this is the first report from Malaysia and the novelty of data represents one of the strengths of the present study.

Inflammation is a mechanism of the innate immune system which provides the host’s first line of defense against infections in a non-specific manner. Development of CRC has been strongly associated with innate immune processes and intestinal inflammation. In CRC, inflammation which can be evident at the earliest stage of tumour progression has been implicated in, to act as a pathological incident capable of transforming the incipient tumours into full blown malignancy (Qian and Pollard, 2010; Wogan et al., 2012). Cytokines play a vital role in the mediation and regulation of immune response including tumour promoting inflammation. Many of these cytokines are pro-inflammatory and anti-inflammatory and have been implicated in tumorigenic process (Dranoff, 2004). Genes encoding the cytokines are genetically polymorphic and polymorphisms in inflammatory response genes have been implicated in susceptibility to inflammatory diseases as well as cancers including CRC. 

We observed that *TNF-α *-308AA homozygous variant genotype was associated with a significantly increased risk of CRC compared with -308GG genotype (OR 5.484, 95% CI 2.327 – 12.924, p<0.001). The strong association observed suggest that *TNF-α* -308 G>A polymorphism could be contributing significantly for CRC predisposition risk in Malaysian population and also implicates its role in inflammation mediated colorectal carcinogenesis pathway. Because transcription of *TNF-α* is regulated by the promoter region of the *TNF-α* gene, polymorphisms located in this region could regulate TNF-α production and thus affect the risk of cancer. Compared to the common G allele, the A allele has been reported to increase gene transcription and produce a six-to seven fold higher level of *TNF-α* transcription (Al-Meghaiseeb et al., 2016). So it is presumed that the SNPs within the *TNF* such as -308 G>A might be causing structural changes within regulatory sites that could affect the function or regulation of TNF alpha production. The uncontrolled excessive production might be contributing to colorectal carcinogenesis. According to Stokker and Hommes (2004), elevated TNF-α play a pivotal role in the pathogenesis of the inflammatory response by interacting with* TNF* receptors and regulating apoptosis, cell proliferation and differentiation.

Our results are in agreement with few other studies. In a study by Garrity-Park et al (2008), the heterozygous and homozygous variant at *TNF-α* - 308 G>A was demonstrated to be associated with CRC susceptibility. Moreover they also reported that this polymorphism was associated with severity of CRC. Li et al., (2011) also observed a positive association of this polymorphism with CRC susceptibility in Chinese population. A meta-analysis involving 14 studies with a total number of 2,837 CRC cases and 3,601 controls by Min et al., (2014) also indicated that *TNF-α* -308 G>A was moderately associated with an increased risk of colon cancer risk in Western population. However, our results are in disagreement with few other studies. A study by Wu et al., (2008) reported that *TNF-α *-308G>A gene polymorphism was not associated with the development of colorectal cancer, but *TNF- α* -308 A/A genotype and A allele were related to the progression of colorectal cancer in Chinese population. Jang et al., (2001) in Korean population, Landi et al., (2003) in Spanish population, Toth et al (2007) in Hungarian population and Kapitanovic et al (2014) in Croatian population did not find any association of this polymorphism with CRC risk. Few other earlier studies (Theodoropoulos et al., 2006; Ohtani et al., 2009; Theodoratou et al., 2012; Guo et al., 2013; Stanilov et al., 2014) and two earlier meta- analyses (Wang et al., 2011; Chen et al., 2013) also reported no association of *TNF-α* -308 G>A polymorphism with CRC risk. The results from two recent other studies, ([Banday et al (2016) in ethnic Kashmiri (North Indian) population and Hamadien et al., (2016) in Saudi Arabian population)] also indicated that there was no significant association between *TNF-α* -308G>A promoter SNP and the risk of developing CRC. This could be attributed to the difference in the polymorphic allele frequencies of TNF- α -308G>A in different population ethnic groups. 

In conformity with our previous report (Mustapha et al., 2012), the homozygous variant *IL-8* -251AA genotype was associated with a significantly increased risk of CRC as compared with homozygous wild type-251TT genotype (OR 4.133, CI 1.937-8.818, P<0.001). Similarly, in case of allele frequency also, such an association was observed. When compared with wild type allele T of SNP *IL-8* -251, the variant allele A showed significantly increased risk for CRC predisposition with OR 1.345 (95% CI 1062-1.704, p=0.013). The strong association that was observed in the present study in CRC patients prompts to suggest that *IL-8* -251T>A polymorphism could be contributing significantly for CRC risk in Malaysian patients. Disrupted gene expression or altered protein formation of *IL-8* gene may contribute positively or negatively to the establishment or progression of CRC. The relevance of *IL-8*-251 T>A polymorphism in CRC susceptibility could be explained by the functional alteration of gene product. Researchers had suggested that, the properties of *IL-8* might be giving pro-angiogenic effect in the tumour and endothelial cells and enhancing the tumour cell proliferation as well as prolonging the survival of human endothelial cells (Huang et al., 2002; Li et al., 2006). As suggested by Wei et al., (2007) higher promoter activity of *IL-8*-251 T>A might increase expression of *IL-8*, resulting in a high function of *IL-8*, inducing Th1- predominant immune response and leading to more susceptibility to CRC than a low production of *IL-8*. Thus the variant allele may be playing a facilitative role in the development of CRC.

**Table 1:** Distribution of Gender and Ages in Cases and Controls

	CRC n = 280	Controls n = 280	P-value	Total
Gender				
Female	140 (50.00%)	140 (50.00%)	1	280
Male	140 (55.50%)	140 (50.00%)		280
Ages				
≥50 years	234 (83.6 %)	126 (45.0 %)	<0.001^α^	360
<50 years	46 (16.4 %)	154 (55.0 %)		200
Mean Age	53.17	46.47		
(Standard deviation)	(± 7.07)	(± 12.02)		

^α^ P < 0.005, statistically significant

**Table 2:** Genotypes and Allele Frequencies of Inflammation Response Genes in CRC Susceptibility

	Genotype				Allele		
	CRC (%)	Controls (%)	p-Value		CRC (%)	Controls (%)	p-Value
*IL-8 251T>A*							
Wild type-251TT	45 (16.1 %)	62 (22.1 %)	0.087	Wild type (T)	289 (51.6 %)	330 (58.9 %)	
Hetero-251TA	199 (71.0 %)	206 (73.6 %)	0.508	Variant (A)	271 (48.4 %)	230 (41.1 %)	**0.014 ** ^α^
Variant-251AA	36 (12.9 %)	12 (4.3 %)	**<0.001** ^α^				
*TNF- α 08G>A*							
Wild type-308 GG	118 (42.2 %)	151 (53.9 %)	**0.005** ^α^	Wild type (G)	368 (65.7 %)	424 (75.7 %)	
Hetero-308 GA	132 (47.1 %)	122 (43.6 %)	0.398	Variant (A)	192 (34.4 %)	136 (24.3 %)	**<0.001** ^α^
Variant-308AA	30 (10.7 %)	7 (2.5 %)	**<0.001** ^α^				
*ICAM-1 K469E*							
Wild type K469K	152 (54.2 %)	145 (51.8 %)	0.553	Wild type (A)	410 (73.2 %)	400 (71.4 %)	
Hetero K469E	106 (37.9 %)	110 (39.3 %)	0.728	Variant (G)	150 (26.8 %)	160 (28.6 %)	0.504
Variant E469E	22 (7.9 %)	25 (8.9 %)	0.648				
*ICAM-1 R241G*							
Wild type R241R	276 (98.6 %)	280 (100 %)	0.123	Wild type (R)	553 (98.8 %)	560 (100.0 %)	
Hetero R241G	1 (0.3 %)	0 (0 %)	1	Variant (G)	7 (1.2 %)	0 (0.0 %)	**0.015** ^α^
Variant G241G	3 (1.1 %)	0 (0 %)	0.249				
*IL-6 174G>C*							
Wild type -174GG	261 (93.2 %)	255 (91.0 %)	0.346	Wild type (G)	539 (96.3 %)	535 (95.5 %)	
Hetero -174GC	17 (6.1 %)	25 (9.0 %)	0.261	Variant (C)	21 (3.7 %)	25 (4.5 %)	0.547
Variant -174CC	2 (0.7 %)	0 (0 %)	0.499				
*PPAR-γ 34C>G*							
Wild type 34CC	274 (97.8 %)	280 (100 %)	**0.030** ^α^	Wild type (C)	554 (98.8 %)	560 (100.0 %)	
Hetero 34CG	6 (2.2 %)	0 (0 %)		Variant (G)	6 (1.2 %)	0 (0.0 %)	**0.031** ^α^
Variant 34GG	0 (0 %)	0 (0 %)					

^α^ **P < 0.05, statistically significant**

**Table 3:** Risk Association of Inflammation Response Gene Polymorphisms with CRC Susceptibility

	Genotype					Allele			
	CRC	Controls	OR (CI 95%)	p-value		CRC	Controls	OR (CI 95%)	p-value
*IL-8*									
TT	45	62	Reference		T	289	330	Reference	
TA	199	206	1.331 (0.865 – 2.047)	0.193	A	271	230	**1.345 (1.062 – 1.704)**	**0.013** ^α^
AA	36	12	**4.133 (1.937 – 8.818)**	**<0.001** ^α^					
*TNF- α*									
GG	118	151	Reference		G	368	424	Reference	
GA	132	122	1.385 (0.981 – 1.954)	0.638	A	192	136	**1.627 (1.254 – 2.110)**	**<0.001** ^α^
AA	30	7	**5.484 (2.327 – 12.924)**	**<0.001** ^α^					
*ICAM-1*									
K469K	152	145	Reference		A	410	400	Reference	
K469E	106	110	0.919 (0.647 – 1.305)	0.638	G	150	160	0.914 (0.704 – 1.140)	0.502
E469E	22	25	0.839 (0.453 – 1.555)	0.578					
*ICAM-1*									
* R241R*	276	280	Reference		R	553	560	Reference	
R241G	1	0	Na	-	G	7	0	Na	-
G241G	3	0	Na	-					
*IL-6*									
GG	261	255	Reference		G	539	535	Reference	
GC	17	25	0.664 (0.350 – 1.260)	0.21	C	21	25	0.834 (0.461 – 1.508)	0.549
CC	2	0	Na	-					
*PPAR-γ*									
CC	274	280	Reference		C	554	560	Reference	
CG	6	0	Na	-	G	6	0	Na	-
GG	0	0	Na	-					

Na,Not available; ^α^** P < 0.05, statistically significant**

**Table 4:** Risk Association of Inflammation Response Gene Polymorphisms and Gender with CRC Susceptibility

	Female				Male			
	CRC	Controls	OR (CI 95%)	p-value	CRC	Controls	OR (CI 95%)	p-value
*IL-8*								
TT	21	30	Reference		24	32	Reference	
TA	103	108	1.362 (0.733 – 2.532)	0.328	96	98	1.306 (0.717 – 2.379)	0.383
AA	16	2	**11.429 (2.372 – 55.054)**	**0.002** ^α^	20	10	**2.667 (1.057 – 6.728)**	**0.038** ^α^
*TNF- α*								
GG	62	72	Reference		56	76	Reference	
GA	59	59	1.151 (0.705 – 1.879)	0.574	73	60	**1.651 (1.016 – 2.683)**	**0.043** ^α^
AA	9	9	**7.661 (2.166 – 27.098)**	**0.002** ^α^	11	4	**3.732 (1.129 – 12.333)**	**0.031** ^α^
*ICAM-1*								
K469K	76	72	Reference		76	73	Reference	
K469E	55	59	0.883 (0.542 – 1.440)	0.618	51	51	0.961 (0.580 – 1.590)	0.875
E469E	9	9	0.947 (0.356 – 2.521)	0.914	13	16	0.780 (0.351 – 1.736)	0.543
*ICAM-1*								
R241R	138	140	Reference		138	140	Reference	
R241G	1	0	Na	-	0	0	Na	-
G241G	1	0	Na	-	2	0	Na	-
*IL-6*								
GG	128	130	Reference		133	125	Reference	
GC	10	10	1.016 (0.409 – 2.523)	0.973	7	15	0.439 (0.173 – 1.111)	0.082
CC	2	0	Na	-	0	0	Na	-
*PPAR-γ*								
CC	136	140	Reference		138	140	Reference	
CG	4	0	Na	-	2	0	Na	-
GG	0	0	Na	-	0	0	Na	-

Na, Not available; ^α^** P < 0.05, statistically significant**

**Table 5:** Risk Association of Inflammation Response Gene Polymorphisms and Age with CRC Susceptibility

	Age < 50				Age > 50			
	CRC	Controls	OR (95% CI)	P-value	CRC	Controls	OR (95% CI)	P-value
*IL-8*								
TT	6	34	Reference		39	28	Reference	
TA	30	113	1.504 (0.578 – 3.916)	0.403	169	93	1.305 (0.755 – 2.256)	0.341
AA	10	7	**8.095 (2.209 – 29.660)**	**0.002** ^α^	26	5	**3.733 (1.276 – 10.919)**	**0.016** ^α^
*TNF-α*								
GG	18	79	Reference		100	72	Reference	
GA	25	69	1.590 (0.800 – 3.159)	0.185	107	53	1.454 (0.929 – 2.274)	0.101
AA	3	6	2.194 (0.501 – 9.615)	0.297	27	1	**19.440 (2.582 – 146.367)**	**0.004** ^α^
*ICAM-1*								
K469K	19	84	Reference		133	61	Reference	
K469E	23	58	1.753 (0.876 – 3.508)	0.113	83	52	0.732 (0.462 – 1.160)	0.184
E469E	4	12	1.474 (0.428 – 5.074)	0.539	18	13	0.635 (0.293 – 1.379)	0.251
*ICAM-1*								
R241R	46	154	Reference		230	126	Reference	
R241G	0	0	Na	-	1	0	Na	-
G241G	0	0	Na	-	3	0	Na	-
*IL-6*								
GG	43	143	Reference		218	112	Reference	
GC	3	11	0.907 (0.242 – 3.400)	0.885	14	14	0.514 (0.237 – 1.115)	0.092
CC	0	0	Na	-	2	0	Na	-
*PPAR-γ*								
CC	46	154	Reference		228	126	Reference	
CG	0	0	Na	-	0	0	Na	-
GG	0	0	Na	-	0	0	Na	-

Na, Not available; ^α^** P < 0.05, statistically significant**

**Table 6:** Risk Association of Inflammation Response Gene Polymorphisms and Smoking Status with CRC Susceptibility

	Smoking				No Smoking			
	CRC	Controls	OR (CI 95%)	p-value	CRC	Controls	OR (CI 95%)	p-value
*IL-8*								
TT	29	50	Reference		16	12	Reference	
TA	120	159	1.301 (0.777 – 2.178)	0.317	79	47	1.261 (0.549 – 2.894)	0.585
AA	17	8	**3.664 (1.407 – 9.538)**	**0.008** ^α^	19	4	3.562 (0.959 – 13.237)	0.058
*TNF- α*								
GG	62	119	Reference		56	32	Reference	
GA	81	92	**1.690 (1.101 – 2.593)**	**0.016** ^α^	51	30	0.971 (0.519 – 1.817)	0.928
AA	23	6	**7.358 (2.847 – 19.014)**	**0.001** ^α^	7	1	4.000 (0.471 – 33.992)	0.204
*ICAM-1*								
K469K	89	115	Reference		63	30	Reference	
K469E	64	88	0.940 (0.615 – 1.437)	0.774	42	22	0.909 (0.463 – 1.785)	0.782
E469E	13	14	1.200 (0.537 – 2.681)	0.657	9	11	0.390 (0.146 – 1.041)	0.06
*ICAM-1*								
R241R	164	217	Reference		112	63	Reference	
R241G	0	0	Na	-	1	0	Na	-
G241G	2	0	Na	-	1	0	Na	-
*IL-6*								
GG	152	199	Reference		109	56	Reference	
GC	12	18	0.873 (0.408 – 1.867)	0.726	5	7	0.367 (0.111 – 1.209)	0.099
CC	2	0	Na	-	0	0	Na	-
*PPAR-γ*								
CC	161	217	Reference		113	63	Reference	
CG	5	0	Na	-	1	0	Na	-
GG	0	0	Na	-	0	0	Na	-

Na, Not available; *, statistically significant

The present study result on the impact of *IL-8*-251 T>A polymorphism in CRC risk in Malaysian patients is in agreement with another case- control study on American population done by Gunter et al., (2006) in which variant genotype *IL-8* -251 AA showed significantly higher risk for CRC predisposition. This has been confirmed by a study by Lurje et al., (2008). Another study on interleukin genes and associations with colon and rectal cancer risk and overall survival showed SNPs from genes within the *IL-8* pathway (*IL8*, *CXCR1* and *CXCR2*) to have significant association with both colon and rectal cancer risk (Bondurant et al., 2013). In contradictory to the present study, Landi et al., (2003) in Spanish population, Theorodopolous et al., (2006) in Greek population and Dumitrescu et al., (2012) in Romanian population reported that, the *IL-8* -251 T>A genotypes was not associated with a higher risk for CRC predisposition. Hu et al., (2012) conducted a meta-analysis including 9 case-control studies with 7003 individuals (3,019 cases and 3,984 controls) focusing on this polymorphism and CRC susceptibility. Their results also concluded that *IL-8* -251 T>A polymorphism was not associated with CRC risk. However, few other meta-analyses on the association of this polymorphism with other cancers showed a positive association with some of the cancers. An association of this polymorphism was reported by Cheng et al., (2013) for gastric cancer risk in Asian population, for Oral cancer risk in Caucasian population by Wang et al., (2013) and for renal cell cancer risk by Chen et al., (2016) in Chinese Han population. In a meta-analysis on association between *IL-8* -251 T>A and risk of lung cancer done by Gao et al., (2014), the results showed that the *IL-8*– 251A/T polymorphism was associated with lung cancer susceptibility in Asians and the –251 A allele may increase risk of lung cancer in Asians.

For the* ICAM-1* K469E polymorphism, there was no significant difference in the variant allele and genotype frequencies between cases and controls. With regard to* ICAM-1* G241R polymorphism, the frequencies of homozygous wild type genotype were significantly higher in controls compared to cases. Surprisingly no heterozygous and homozygous variant genotypes of *ICAM-1* G241R were observed in controls whereas cases showed a low frequency of heterozygous and homozygous variant genotypes. It seems that the variant allele is very infrequent in Malaysian population. In a study by Wang et al., (2009), only the GG genotype was observed in the tested Chinese population, which was consistent also with Japanese and Korean populations respectively, which probably reflects a common ancestor in these populations (Nishimura et al., 2000). On examining the associated risk, the variant genotype of *ICAM-1* K469E, in the present study did not show any significant association for CRC susceptibility risk. For *ICAM-1* G241R, the risk association could not be evaluated in view of the absence of variant allele and genotypes among the controls. Several studies have investigated the relationship between *ICAM-1* gene polymorphisms and tumour susceptibility, but results showed inconsistency. Our results are also in agreement with few studies as well as in disagreement with few other studies. Wang et al’s (2014), meta-analysis involving 14 case-control studies (consisting of a total of 4,358 cases and 5,017 controls) did not support the *ICAM-1* K469E polymorphism as a risk factor for cancer. Cheng and Liang (2015) who conducted a meta-analysis (involving 18 case-control studies with 4,844 cancer cases and 5,618 healthy controls) suggested that *ICAM-1* G241R polymorphism might be a genetic risk factor for development of cancer in general, especially in European population whereas K469E polymorphism might not act as a cancer risk factor. Another meta-analysis by Tan et al., (2015) showed *ICAM-1* K469E polymorphism contributed to decreased susceptibility to cancer in Caucasians, especially for melanoma and CRC, but may be risk factor for oral cancer in Asians. ICAM-1 K469E polymorphism has been associated with increased risk of gastric cancer (Tian et al., 2012).The present study results on *ICAM-1* K469E polymorphism is in disagreement with another study by Theodoropoulos et al., (2006) in Greek population. Their study showed that variant allele of *ICAM-1* K469E was associated with significantly higher risk for CRC predisposition. 

In this study, comparison of the frequencies of alleles and polymorphic genotypes of *IL-6 *-174 G>C in cases and controls, showed no statistically significant difference. The risk was examined for heterozygous variant of *IL-6 *-174G>C only, as homozygous variant was observed in only two CRC patients and none of the controls. However, heterozygous variant did not show any risk association with CRC susceptibility. With the observed results from the present study, it is reasonable to suggest that polymorphism -174 at *IL-6 *gene with change of Guanine to Cytosine may be not associated with CRC predisposition risk in Malaysian population. On the contrary, in Landi et al’s., (2003) study on Spanish population, this SNP showed an increased risk for CRC predisposition (OR 1.55, 95%, CI 1.12-2.09, p=0.0073). Similarly, in the study done by Theodoropoulus et al., (2006), the SNP at promoter region of *IL-6* -174 was found to be associated with increased risk of CRC susceptibility with OR 2.10 (95% CI 1.40-3.16, p=<0.0005) in Greek population. In the same study, the allele frequency of variant allele of *IL-6 *-174 G>C also showed significantly higher risk for CRC predisposition risk with OR 1.77 (95% CI 1.34 – 2.34, p=<0.0001). A meta-analysis by Hu et al (2013) involving 11 populations incorporating 6,481 cases and 7,935 controls, found lack of association between *IL-6*-174 G<C polymorphism and CRC risk. Another meta-analysis by by Zhou et al (2014) also did not find any significant association of* IL-6*-174 G>C polymorphism with CRC risk. Our results are in agreement with results from the above two meta-analyses.

For the SNP 34 C>G of *PPAR-γ* also, none of the patients and controls showed homozygous variant genotype in the present study, while heterozygous variant was observed in 2.2% of CRC patients and none in controls. When the genotype frequencies were compared between cases and controls, the homozygous wild type was significantly higher in controls (0.030). Likewise, the frequencies of major allele was found to be higher in controls (p=031). It seems that the variant allele is infrequent in Malaysian population. Because of this same reason, the risk association of *PPAR-γ* could not be determined and *PPAR-γ* polymorphism singly, does not seem to be contributing in CRC susceptibility risk in this population. In a previous study on Greek population, Theorodopoulos et al., (2006) reported that, both variant allele and genotype frequencies of *PPAR-γ *34 C>G reduced the risk for CRC susceptibility. Study by Gong et al., (2005) and Landi et al., (2003) also reported that, *PPAR-γ* was associated with reduced risk of CRC predisposition. Sarraf et al., (1999) reported that *PPAR-γ *contributed to suppression of colon cancer. A study by Gong et al., (2005) showed that *PPAR-γ* 34 C>G was associated with reduced risk colorectal adenoma in USA population. Another study by Koh et al (2006) on *PPAR-γ *34 C>G polymorphism and CRC risk among Chinese in Singapore also showed lower risk. Lu et al., (2010), in a meta-analysis comprising of 9 studies with a total number of 4,533 cases and 6,483 controls reported that *PPAR-γ *34 C>G was associated with colon cancer risk, but not associated with rectal cancer risk. Another recent meta-analysis based on 15 studies including 13,575 cases and 17,085 controls by Wei et al., (2015) also found that *PPAR-γ* 34 C>G polymorphism was associated with a reduced risk of CRC in Caucasians and no significant association with CRC risk in Asians. 

As additional exploratory analysis, the association between genotype frequency and CRC risk was investigated after stratifying the subjects based on gender, age and smoking habits. When stratified according to gender, the variant genotypes of *IL-8* and *TNF-α* showed a higher risk for CRC among females. The underlying reason for this gender bias is still unknown. The immune and inflammatory response between males and females might be different. It is presumed that genetic factors may at least, in part account for gender-related differences in immune / inflammation response. Age wise stratification showed that carriers of *TNF-α* AA genotype with 50 years and above had a significantly higher risk for CRC susceptibility. This is quite expected because CRC is a disease associated with old age. But the variant genotype AA of *IL-8* was associated with a higher risk for CRC in both groups aged < 50 years and > 50 years, suggesting its influence on inflammation mediated CRC risk among all age groups. 

The modifying effect of smoking habit on association between inflammatory response gene polymorphisms and CRC risk was evaluated by comparison of results for smokers and non-smokers. A significant interaction was observed between smoking habits and *IL-8* and *TNF-α* polymorphisms. It has been well documented that cigarette smoking is one of the life-style factors that play an important role in the exposure of an individual to the carcinogens present in cigarette smoke. Various carcinogens such as polycyclic aromatic hydrocarbons, heterocyclic amines, nitroso-amines and aromatic amines, present in the cigarette smoke can cause harm to the human colon and rectum through formation of DNA adducts (Giovannucci, 2004). Studies (Stern et al., 2007; Wu et al., 2004) have demonstrated that these carcinogens, which can reach the colorectal mucosa through direct ingestion or via the circulatory system induce bulky adducts in cryptic cells and contribute to the formation of mutations in the colon. So, cigarette smoking will have a synergistic effect on the colorectal tissues which also promote colorectal carcinogenesis. 

From the results, it is reasonable to presume that SNPs showing higher risk might be promoting CRC susceptibility by altering the properties of cytokines and chemokines they encode and acting as negative regulators of cellular growth. According to Theodoropoulus et al., (2006) inflammation favours tumorigenesis by stimulating angiogenesis, damaging DNA and chronically stimulating DNA proliferation. In a continuous inflammatory condition, the inflammatory cells will produce reactive oxygen species (ROS) and reactive nitrogen species (RNS). Cancer development occur when inflammatory cells initiate the production of ROS and/or RNS in inflamed tissues and will cause DNA damage which will lead to activation of oncogenes and/or inactivation of tumor suppressor genes (Kundu and Surh, 2008). Hence, it is reasonable to suggest that, in a continuous inflammatory condition, genetic variation in immune and inflammatory response genes leading to aberrant response of host’s immune system, may be acting as genetic predisposition factors, favouring inflammation mediated colorectal carcinogenesis. 

Polymorphisms which showed conflicting results in different populations might be attributable to the presence of genetic heterogeneity across ethnic populations, small sample size limitations and difference in methodologies employed. Another reason could be pleiotropic role of the polymorphism in the pathogenesis of CRC, or due to interaction with other genetic and environmental factors. Yet another explanation could be linkage disequilibrium (LD) patterns. A polymorphism may be in LD with another nearby causal variant in one ethnic population but not in another. Some variants may have stronger or weaker effects on disease, depending on environment or genetic background. Since CRC risk is determined by complex interactions between various genetic and lifestyle/dietary risk factors, common genetic variants are likely to interact with these environmental – lifestyle risk factors to modify risk.

In conclusion, we observed an association between variant allele and genotypes of *IL-8*-251 T>A and *TNF-α*-308 G>A polymorphisms and CRC risk in Malaysian patients. These two SNPs in inflammatory response genes which undoubtedly contribute to individual risks to colorectal cancer susceptibility could be considered as potential genetic predisposition factors. Results are also in favour of an inflammation mediated pathway for colorectal carcinogenesis. Although the findings presented here are exploratory, our study is limited by its small sample size. In view of our findings as exploratory, and given the emerging evidence on chronic inflammation and CRC risk, further rigorous studies are necessary to resolve various complications and elucidate missing links between inflammation and colorectal cancer.
